# Pregnane X Receptor and Yin Yang 1 Contribute to the Differential Tissue Expression and Induction of CYP3A5 and CYP3A4

**DOI:** 10.1371/journal.pone.0030895

**Published:** 2012-01-23

**Authors:** Dieudonné Nem, Dorothea Baranyai, Huan Qiu, Ute Gödtel-Armbrust, Sebastian Nestler, Leszek Wojnowski

**Affiliations:** Department of Pharmacology, University Medical Center, Johannes Gutenberg University Mainz, Mainz, Germany; Ecole Normale Supérieure de Lyon, France

## Abstract

The hepato-intestinal induction of the detoxifying enzymes CYP3A4 and CYP3A5 by the xenosensing pregnane X receptor (PXR) constitutes a key adaptive response to oral drugs and dietary xenobiotics. In contrast to CYP3A4, CYP3A5 is additionally expressed in several, mostly steroidogenic organs, which creates potential for induction-driven disturbances of the steroid homeostasis. Using cell lines and mice transgenic for a *CYP3A5* promoter we demonstrate that the CYP3A5 expression in these organs is non-inducible and independent from PXR. Instead, it is enabled by the loss of a suppressing yin yang 1 (YY1)-binding site from the *CYP3A5* promoter which occurred in haplorrhine primates. This YY1 site is conserved in *CYP3A4*, but its inhibitory effect can be offset by PXR acting on response elements such as XREM. Taken together, the loss of YY1 binding site from promoters of the *CYP3A5* gene lineage during primate evolution may have enabled the utilization of CYP3A5 both in the adaptive hepato-intestinal response to xenobiotics and as a constitutively expressed gene in other organs. Our results thus constitute a first description of uncoupling induction from constitutive expression for a major detoxifying enzyme. They also suggest an explanation for the considerable tissue expression differences between CYP3A5 and CYP3A4.

## Introduction

Transcriptional activation of metabolizing enzymes and transporters in the small intestine and in the liver constitutes the most important adaptive response to oral drugs and dietary xenobiotics. The involved transcription factors are activated by xenobiotics and are therefore collectively referred to as xenosensors. Due to its wide ligand-binding spectrum, the pregnane X receptor (PXR, NR1I2) is the most important human xenosensor [1]. The Phase I enzyme Cytochrome P450 3A4 (CYP3A4) [1] and its somewhat less substrate-promiscuous paralog Cytochrome P450 3A5 (CYP3A5) [2] belong to the most prominent gene targets induced by PXR. The various reactions catalyzed by CYP3A4 and CYP3A5, most notably oxidations, facilitate Phase II conjugating reactions and thereby the removal of xenobiotics from the body. Substrates of these enzymes include an estimated 50% of contemporary drugs [3], [4].

The protective effects of the hepato-intestinal *CYP3A* induction come at the expense of disturbed homeostatsis of important metabolic processes. This is due to the participation of CYP3A in the metabolism of steroid hormones, bile acids, and retinoids [1]. For example, the anti-tuberculosis drug and specific PXR agonist rifampicin affects vitamin D homeostasis [5], leading to osteomalacia [6]. This is consistent with the involvement of CYP3A4 and CYP3A5 in the hepato-intestinal vitamin D metabolism [7], [8], [9].

The potential of homeostatic disturbances is particularly high for CYP3A5 which, unlike CYP3A4, is expressed in the steroidogenic organs prostate [10], [11], [12], adrenal gland [12], and kidney [12], [13]. The physiological significance of the CYP3A5 expression in these organs is unknown, but could be related to steroid metabolism. For example, the renal CYP3A5 expression level has been associated with salt-dependent hypertension [14], [15]. Besides proximal and distal tubules, CYP3A5 is expressed in the collecting ducts [16], [17], [18], where it is thought to affect the mineralocorticoid-driven sodium reabsorption. The underlying mechanism is incompletely understood but it could involve the mineralocorticoid effect of 6ß-hydroxylated glucocorticoids generated by CYP3A5 [19], [20]. Additionally or alternatively, renal CYP3A5 activity could regulate the glucocorticoid occupancy of mineralocorticoid receptors [21]. Although the renal CYP3A5 expression level is in all likelihood mainly determined by genetic polymorphisms [22], [23], its level in CYP3A5 expressors could be affected by induction, similarly to what has been observed in the liver and small intestine [2]. In addition to influencing endogenous compounds such as steroids, *CYP3A5* induction in the kidney could exert medically important local effects on drug metabolism. This can be inferred from the observation that microsomes derived from CYP3A5-expressing kidneys faster inactivate the immunosuppressive drug tacrolimus. This has been suggested to diminish the intra-organ tacrolimus concentrations in transplanted kidneys, which could accelerate their rejection [24]. CYP3A5-expressing kidneys also generate higher amounts of nephrotoxic metabolites of drugs such as cyclosporine A [25] and the alkylating agent ifosfamide [26].

The above considerations have spurred investigations of the determinants of the CYP3A5 expression in tissues other than liver and small intestine, and of the differential tissue expression of CYP3A5 and CYP3A4 in general. The non-expression of CYP3A4 as opposed to CYP3A5 in a lung-derived cell line has been linked to a 57 bp insertion into the gene's promoter, but the exact mechanism has not been identified [27]. The expression of CYP3A5 in the prostate has been reported to be mediated by a promoter element binding the androgen receptor [10]. No comparable investigations have been reported for the kidney which, somewhat surprisingly, exhibits extremely low or non-detectable expression of *PXR* transcripts [12], [28], [29], [30], [31], [32], [33]. Likewise, presently there is no data on CYP3A5 induction in organs other than liver and small intestine [2].

Therefore, we investigated the determinants of the CYP3A5 expression, initially concentrating on the kidney as a model organ. To this end, we first established a two-cell line model reflecting the expression relationships of CYP3A4 and CYP3A5 in the kidney and small intestine *in vivo*. Our data demonstrate that the CYP3A5 expression in renal cells was enabled by the loss of a suppressing yin yang 1 (YY1)-binding site from the *CYP3A5* promoter. This allowed for a renal, but in all likelihood also adrenal and pulmonary CYP3A5 expression insensitive to PXR induction, as confirmed in *CYP3A5* transgenic mice. The YY1 element is retained in the *CYP3A4* promoter, but its effect is abrogated by PXR acting on response elements such as the xenobiotic-responsive enhancer module (XREM). The differential organ expression and induction of CYP3A4 and CYP3A5 results thus from the loss of the YY1 binding element from the *CYP3A5* promoter, acting in concert with the differential organ expression of PXR, and with the higher accumulation of PXR response elements in the *CYP3A4* promoter.

## Results

### Evaluation of CYP3A5 and CYP3A4 proximal promoter activities in renal and intestinal cells

**Figure 1:**
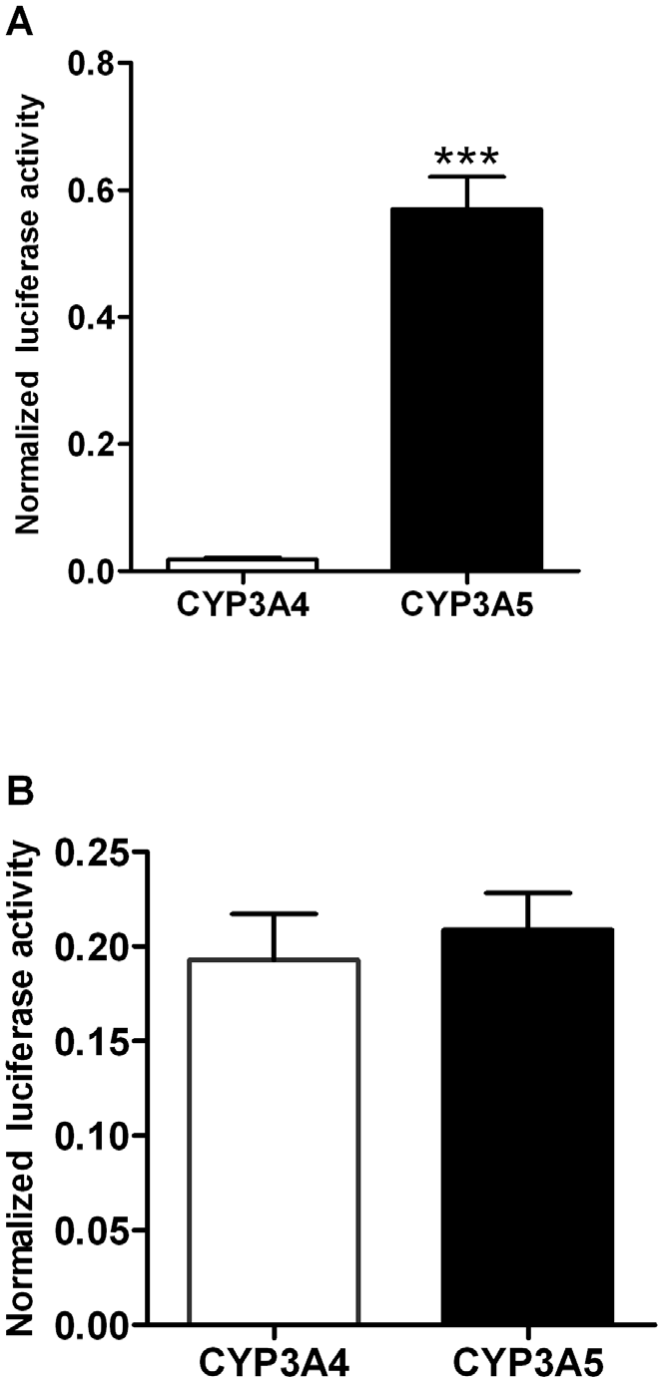
The activities of proximal *CYP3A4* (374 bp) and *CYP3A5* (370 bp) promoters in kidney-derived MDCK.2 cells (A) and in small intestine-derived LS174T cells (B). Data are expressed as mean values (±SEM) of six independent experiments conducted as triplicates. Promoter-driven firefly luciferase activities in the individual wells were normalized using activities of the co-transfected renilla luciferase driven by a constitutive promoter. Statistically significant differences are indicated by asterisks (*** *p*<0.001).

The conservation of the primate *CYP3A5* and *CYP3A4* promoters is limited to their most proximal parts [34]. We investigated if these parts are sufficient to confer the previously reported differential expression of these genes in renal cells [12]. To this end we applied plasmids expressing firefly luciferase under the control of *CYP3A4* and *CYP3A5* proximal promoter fragments of comparable lengths of 374 and 370 bp, respectively. These plasmids were transiently transfected into kidney-derived cell line MDCK.2. These cells exhibit many characteristics of tubular and collecting duct cells [35], [36], which are the site of CYP3A5 expression in humans [16], [17]. The activity of the *CYP3A5* promoter was robust, whereas that of the *CYP3A4* promoter was 31-fold lower (Figure 1A). On the other hand, the activities of these promoters were similar in the small intestine-derived cell line LS174T (Figure 1B). These findings were fully compatible with the expression relationships between *CYP3A4* and *CYP3A5* in the human kidney and in the small intestine *in vivo* [12]. Therefore, these cell lines were taken together as a model for more detailed investigations of the determinants of the differential renal and intestinal CYP3A4 and CYP3A5 expression.

### Function of the 57 bp difference between the CYP3A4 and CYP3A5 promoters

**Figure 2:**
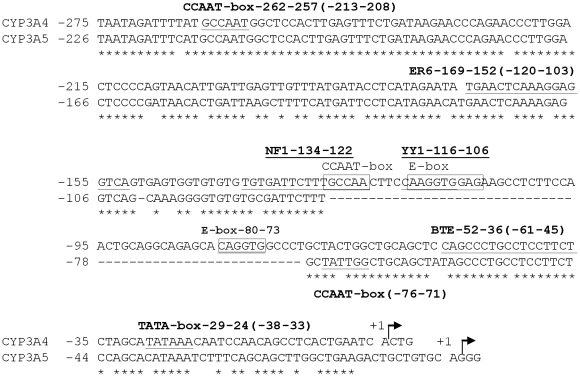
Sequence comparison and distribution of regulatory elements in the human *CYP3A4* and *CYP3A5* proximal promoters. Identical nucleotides are denoted by asterisks. The 57 bp region absent from the *CYP3A5* promoter is represented as a stretch of hyphens. The transcription start sites [37], [38] are indicated by arrows. The sequence is numbered relative to the transcription start site taken as +1. The binding sites for previously characterized transcriptional regulators CCAAT-box, ER6, BTE, TATA-box, and NF1 [37], [38], [39] are underlined. The portion of the NF1 binding site described to constitute a CCAAT box, the YY1 site, and the two E-box motifs [27], [39], all contained in the 57 bp region, are boxed. The positions of binding sites are shown separately for *CYP3A4* and, if applicable, in brackets for *CYP3A5*.

**Figure 3:**
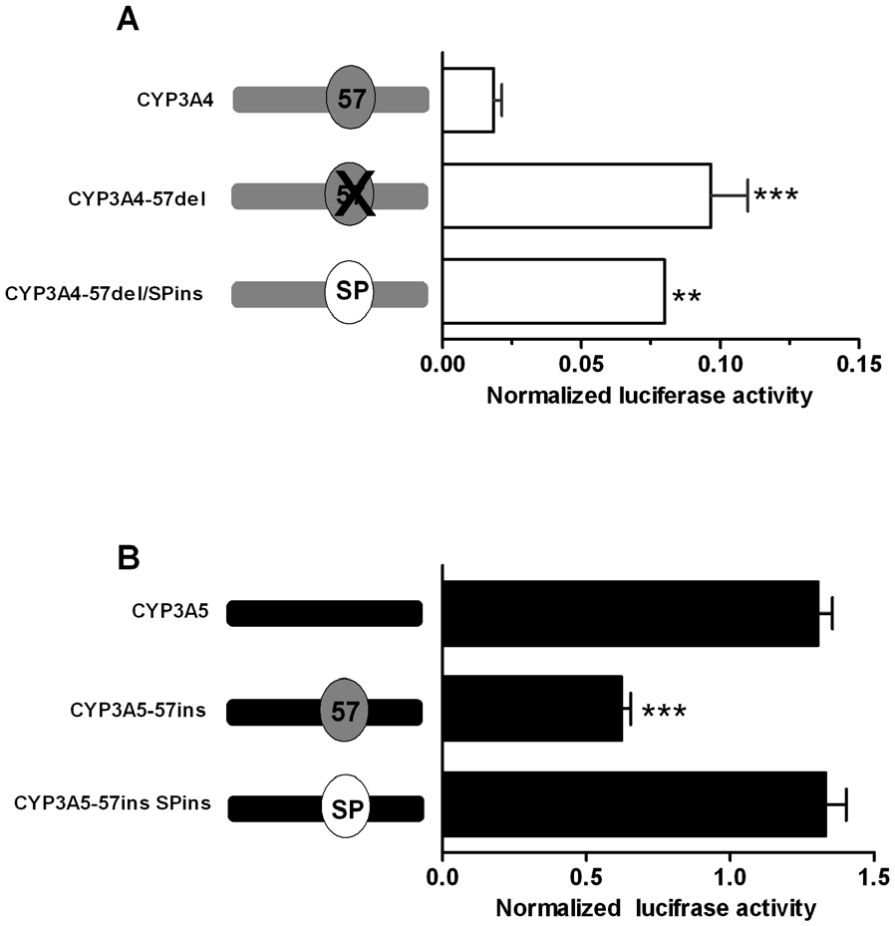
The effect of the *CYP3A4*-derived 57 bp region on the activities of the proximal *CYP3A4* and *CYP3A5* promoters in MDCK.2 cells. (**A**) The effect of a deletion of the 57 bp region from the proximal *CYP3A4* promoter, or of its replacement with an unrelated “spacer” (SP) sequence of identical length. (**B**) The effect of the insertion of the 57 bp region, or of the “spacer” into the *CYP3A5* promoter. Data are expressed as mean values (± SEM) of three to six independent experiments conducted as triplicates. Promoter-driven firefly luciferase activities in the individual wells were normalized using activities of the co-transfected renilla luciferase driven by a constitutive promoter. Statistically significant differences are indicated by asterisks (** *p*<0.01,*** *p*<0.001).

The most prominent difference between the proximal *CYP3A5* and *CYP3A4* promoter sequences is the presence of a 57 bp fragment in *CYP3A4* which is absent from *CYP3A5*. This region is localized upstream of the basic regulatory elements: the CCAAT-box, the basic transcription element (BTE), the TATA-box, and downstream of the everted repeat separated by 6 base pairs (ER6) and the nuclear factor 1 (NF1) enhancer elements (Figure 2), which have been characterized in previous studies [37], [38], [39]. Except NF1, all these elements are conserved between the *CYP3A4* and *CYP3A5* promoters. To determine the role of the 57 bp fragment in the absence of *CYP3A4* expression in renal cells, it was deleted from the proximal *CYP3A4* promoter. In parallel, this sequence was replaced by one of identical length but with no apparent transcriptional activity (“spacer”, SP in Figure 3). By using a spacer we wanted to detect *CYP3A4* promoter activity changes independent from the content of the 57 bp fragment, but related to any altered spatial interactions among surrounding *cis*-acting elements following its deletion. Conversely, the *CYP3A4*-derived 57 bp region, or alternatively the spacer, was inserted into the corresponding location in the *CYP3A5* promoter. The resulting constructs (*CYP3A4*-57del, *CYP3A4*-57del/SPins, *CYP3A5*-57ins, and *CYP3A5*-SPins, respectively) were assessed for activity in MDCK.2 cells in parallel to the corresponding wild-type promoters. The deletion of the 57 bp element increased the activity of the *CYP3A4* promoter 4-fold (Figure 3A). The replacement of the 57 bp fragment with a spacer (*CYP3A4*-57del/SPins construct) had a similar effect (Figure 3A). Conversely, the *CYP3A5*-57ins construct exhibited a ∼2/3 decrease in the luciferase activity in comparison to the wild-type *CYP3A5* promoter (Figure 3B), whereas no such effect was observed following the spacer insertion.

### Evolutionary history of the 57 bp region in primates

**Figure 4:**
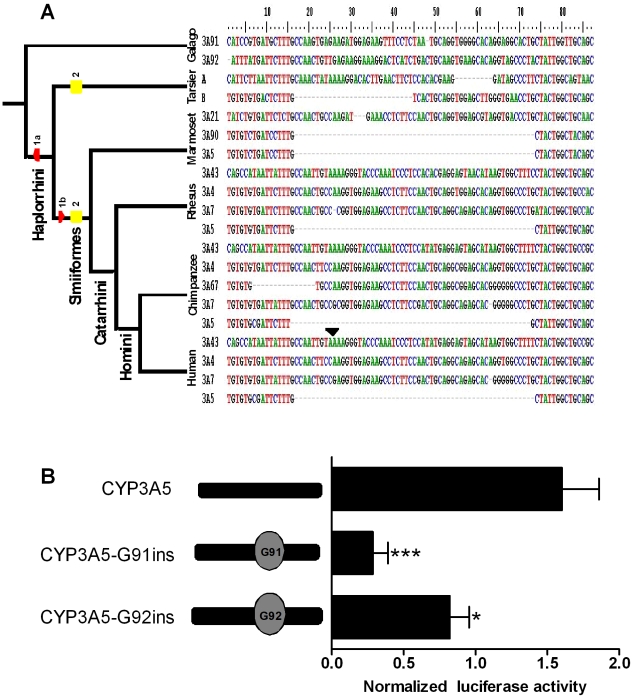
Genomic and functional conservation of the 57 bp *CYP3A* promoter region in primates. (A) Representation of the evolution of the 57 bp region. Deletions are shown as stretch of hyphens, with the widest one corresponding to the deletion of the entire 57 bp region. 1a–b and 2 indicate the two alternative scenarios of the 57 bp deletion. “CYP” has been removed from gene names to improve legibility. A 7 bp fragment present only in all *CYP3A43* genes has been removed for clarity and it position in the human CYP3A43 gene is indicated by an arrow. (B) The effect of galago CYP3A91- and CYP3A92-derived 57 bp regions on the human CYP3A5 promoter activity in MDCK.2 cells. Data are expressed as mean values (±SEM) of five independent experiments conducted as triplicates. Promoter-driven firefly luciferase activities in the individual wells were normalized using activities of the co-transfected renilla luciferase driven by a constitutive promoter. Statistically significant differences are indicated by asterisks *p<0.05, ***p<0.001.

The above data demonstrated that the 57 bp fragment contained elements repressing the activity of *CYP3A* promoters in renal cells. In order to identify the responsible mechanism, the 57 bp region was investigated in more detail *in silico* and *in vitro*. To illuminate its evolutionary history and to increase the specificity of the predicted regulatory elements, we first searched for homolog sequences from several primate species. Homolog sequences were found in both galago *CYP3A* genes (*CYP3A91* and *CYP3A92*), in both tarsier *CYP3A* genes, provisionally designated by us as gene *A* and *B* (cont323625 and contig840032 of the genomic assembly tarSyr1), in the *CYP3A21* of the marmoset, as well as in all *CYP3A4*, *CYP3A7*, and *CYP3A43* genes from rhesus, chimpanzee, and human (Figure 4A). Furthermore, sequences ortholog to the 57 bp fragment were identified in many non-primate mammalian *CYP3A* genes (data not shown). In contrast, we found the 57 bp fragment fully deleted from the promoters of all primate *CYP3A5* genes (Figure 4A). In addition, a partial deletion of the most distal 25 bp within the 57 bp fragment was found in the tarsier gene *B*. To verify if the repressive effect of the 57 bp region is conserved in primates, ortholog sequences derived from the galago genes *CYP3A91* and *CYP3A92* were inserted into the human *CYP3A5* proximal promoter. Sequence from either gene repressed the luciferase activity in renal cells.

### The 57 bp region contains a conserved YY1-binding site

**Figure 5:**
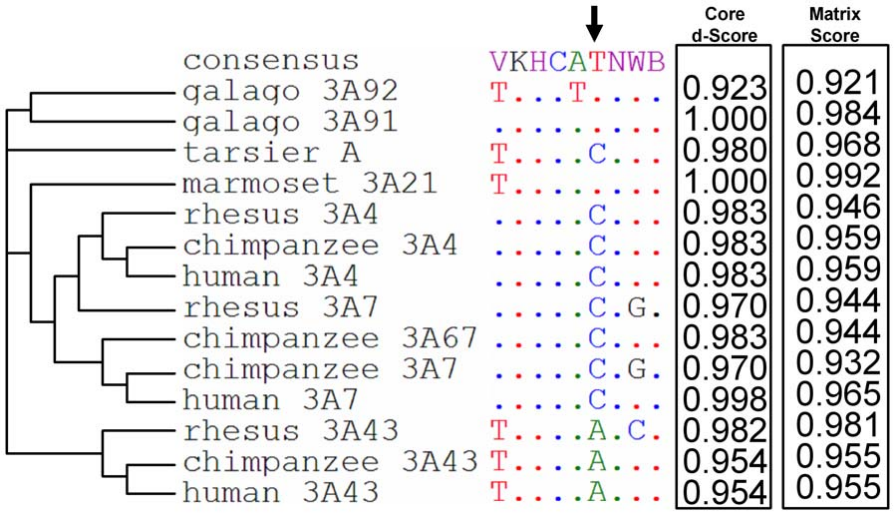
Conservation and P-Match scores of the YY1 site in the 57 bp fragment of primate *CYP3A* promoters. The 5′-VKHCATNWB-3′ consensus for YY1 is depicted on the top of sequences in the IUB code. Nucleotides identical to the equivalence consensus are denoted by dots. The arrow indicates the T>C mutation in the YY1 core motif. The phylogenetic tree of selected primate *CYP3A* genes on the left was adopted from a previous study [68]. “*CYP*” has been removed from gene names to improve legibility.

Besides a portion of the NF1-binding element and an E-box motif, the 57 bp fragment contains on the anti-parallel strand a binding site for a dual-function transcriptional regulator yin yang 1 (YY1) (Figure 2). YY1 binding to this element in the human *CYP3A4* promoter had been reported previously [39], but its functional significance was unknown. Considering the established role of YY1 as a transcriptional repressor, we concentrated on the binding site for this protein. YY1 is known to bind to a highly degenerated consensus sequence 5′-(C/g/a)(G/t)(C/t/a)**CAT**N(T/a)(T/g/c)-3′ with uppercase and lowercase letters representing the preferred and tolerated nucleotides, respectively. The bolded tri-nucleotide **CAT** constitutes the YY1 binding core motif [40]. The highest concordance with the consensus sequence was found in galago *CYP3A91* and in marmoset *CYP3A21*, which was reflected by the highest P-Match score values (Figure 5). In contrast, all human, chimpanzee, and rhesus *CYP3A4* and *CYP3A7* promoters, as well as the promoter of the chimpanzee-specific *CYP3A67* gene and of the tarsier *A* gene contain the mismatch T>C in the core motif CAT which is accompanied by decreased core d-scores. The higher score values of *CYP3A91*, as compared to *CYP3A92*, were in agreement with the stronger *in vitro* effects of the 57 bp insert derived from the former gene (Figure 4B).

### Functional characterization of the human CYP3A4 YY1 binding site

**Figure 6:**
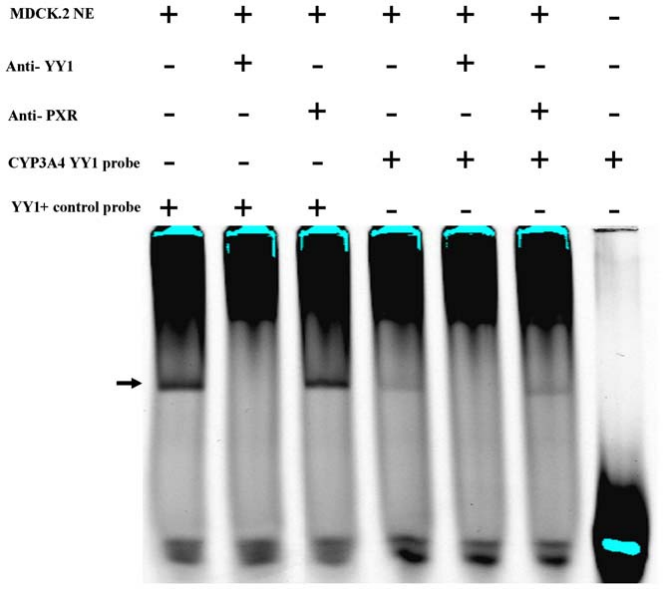
Binding of YY1 to the 57 bp element of the *CYP3A4* promoter. Electrophoretic mobility shift assay of oligonucleotides containing the *CYP3A4*-derived YY1 binding sequence incubated with an MDCK.2 cells-derived nuclear extract (NE; 20 µg). The rpL30 gene-derived oligonucleotide containing an unrelated, previously described [41] YY1 binding site was used as a positive control. Reactions contained (+) or lacked (−) the indicated component. The arrow points to the YY1-DNA-binding complex. The immunodepletion was achieved with an anti-YY1 antibody (1 µg). An anti-PXR antibody (300 ng) was used as a negative control.

We first confirmed the reported binding of YY1 to the consensus binding site within the *CYP3A4* promoter-derived 57 bp fragment [39] using an electrophoretic mobility gel shift assay (EMSA). An IRDye800-labeled oligonucleotide encompassing the *CYP3A4* YY1 binding site served as a probe. A previously described YY1-binding sequence from an unrelated gene [41] was included as a positive control. A shifted complex was obtained for the *CYP3A4* YY1 region-derived oligonucleotide with MDCK.2 cell-derived nuclear extract (Figure 6). The complex migrated at the same level as the YY1-DNA positive control complex. The identity of the shift was confirmed with an anti-YY1 antibody, which resulted in an immonodepletion. In contrast, an anti-PXR antibody, included as a negative control, had no effect.

**Figure 7:**
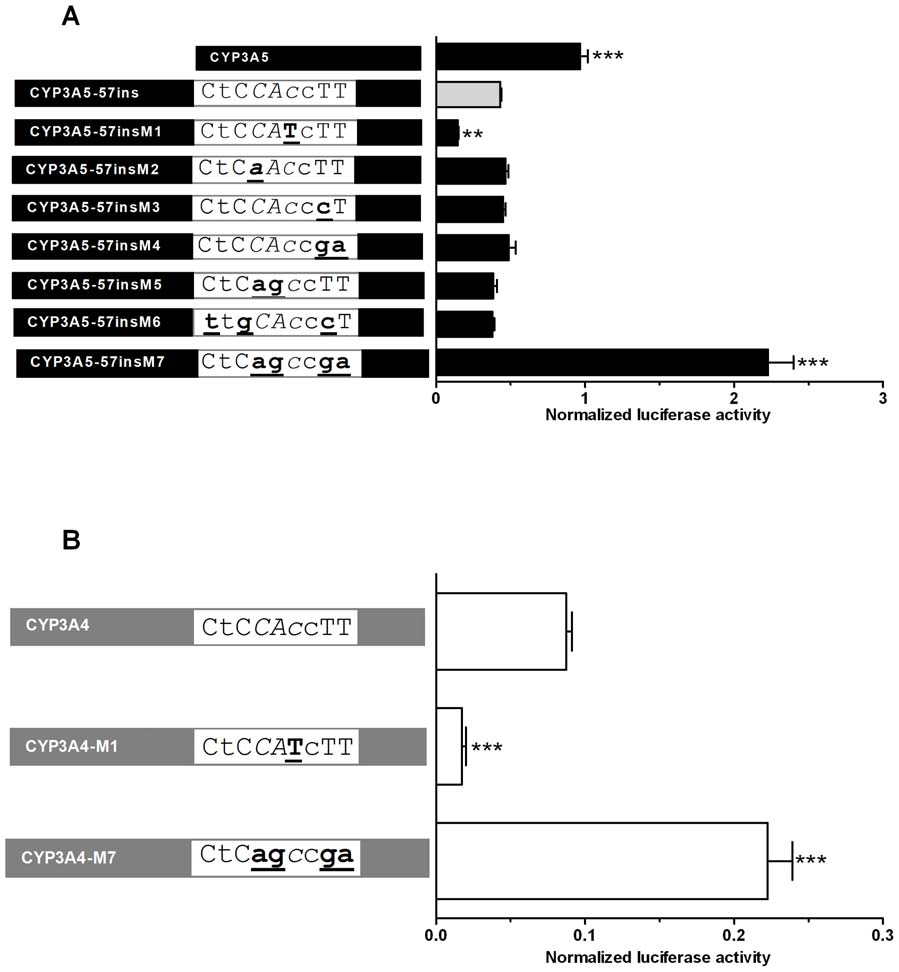
Mutational analysis of the *CYP3A4*-derived YY1 binding site expressed (A) in MDCK.2 cells in the *CYP3A5-57ins* promoter construct and (B) in LS174T cells in the native *CYP3A4* promoter. The uppercase and lowercase letters represent preferred and tolerated nucleotides, respectively. The bolded and underlined letters indicate the mutated nucleotides. The mutations either restore the consensus core motif (M1), or progresively disrupt the YY1 binding site (M2 to M7). The construction of *CYP3A4* and *CYP3A5* mutants is described under “Materials and Methods.” Promoter-driven firefly luciferase activities were normalized using activities of the co-transfected renilla luciferase driven by a constitutive promoter and compared in (A) to that of the *CYP3A5*-ins57 construct and in (B) to that of the wild-type *CYP3A4*. Data are expressed as mean values (±SEM) of four to eight independent experiments, conducted as triplicates. Statistically significant differences are indicated by asterisks (** p<0.01,*** p<0.001).

The functional importance of the *CYP3A4*-derived YY1 binding site was then investigated in the *CYP3A5* promoter context (*CYP3A5*-57ins construct from Figure 3B) using mutagenesis followed by transfection into MDCK.2 cells. Statistically significant effects were observed with two mutants: The *CYP3A5*-57insM1 mutant converts the imperfect YY1 core motif CAC into a consensus motif CAT such as seen in galago *CYP3A91* and in marmoset *CYP3A21*. This enhanced the repression of the promoter activity conferred by the 57 bp fragment (Figure 7A). In the *CYP3A5*-57insM7 mutant, the core motif consensus dinucleotide CA was replaced with the non-consensus dinucleotide AG. Simultaneously, the dinucleotide TT outside the binding core motif, implicated in the specificity of YY1 binding [42], was replaced by the dinucleotide GA. This mutant not only fully abolished the repressive effect of the 57 bp region on the *CYP3A5*-driven luciferase but increased its activity 5-fold in comparison to the wild-type *CYP3A5* promoter (Figure 7A). As no such excessive activity was observed with the “spacer” sequence (*CYP3A5*-Spins, Figure 3B), this suggested the existence within the 57 bp fragment of additional, as yet unidentified transcriptional enhancers which come to light after the removal of the YY1-mediated repression. However, mutant *CYP3A5*-57M6, generated to inactivate a putative E-box-like binding site [27] overlapping with the YY1-binding site (Figure 2), had no effect on activity. Likewise, we saw no changes in luciferase activity upon the mutation of the NF1 or of the second, more downstream E-box binding site (Figure S1). Taken together with the EMSA experiments (Figure 6), these results demonstrated the existence of an YY1 binding site within the 57 bp fragment of the *CYP3A4* promoter which mediated transcriptional repression in renal cells. They also suggested the existence of as yet unidentified pro-transcriptional elements in this fragment which are, however, fully suppressed by YY1.

### The effects of YY1 and PXR on the CYP3A4 promoter activity in intestinal cells

The loss of the YY1 response element from the *CYP3A5* promoter and its retention in the *CYP3A4* promoter were fully consistent with the differential expression of these genes in renal cells. However, this mechanism was in an apparent contrast with the expression of CYP3A4 in small-intestinal cells LS174T (Figure 2B) and in the small intestine *in vivo* [12]. We reasoned that the absence of *CYP3A4* repression in intestinal cells was brought about by a mechanism overriding the repressive effect of the YY1 binding element in the *CYP3A4* promoter. In the most parsimonious scenario, this could be achieved by the absence of YY1 expression in intestinal cells. We tested this hypothesis by measuring the expression of YY1 mRNA in either cell line. In agreement with previous reports of an ubiquitous YY1 expression [43] its mRNA was detected both in LS174T and MDCK.2 cells (data not shown). An overexpression of YY1 in LS174T cells approximately halved the luciferase activity driven by the *CYP3A4* promoter (Figure S2). Furthermore, mutations of the YY1 site, tested in LS174T cells in the *CYP3A4* promoter context, showed an identical response profile (Figure 7B and data not shown) as in the *CYP3A5* promoter context in MDCK.2 cells (Figure 7A). Thus, the restoration of the consensus YY1 core motif (*CYP3A4*-M1) significantly reduced, whereas the disruption of the site (*CYP3A4*-M7) increased the *CYP3A4* promoter activity (Figure 7B). Taken together, these result suggested similar effects of YY1 in renal and intestinal cells, arguing against the importance of this factor in the differential expression of CYP3A4 in the kidney and small intestine.

**Figure 8:**
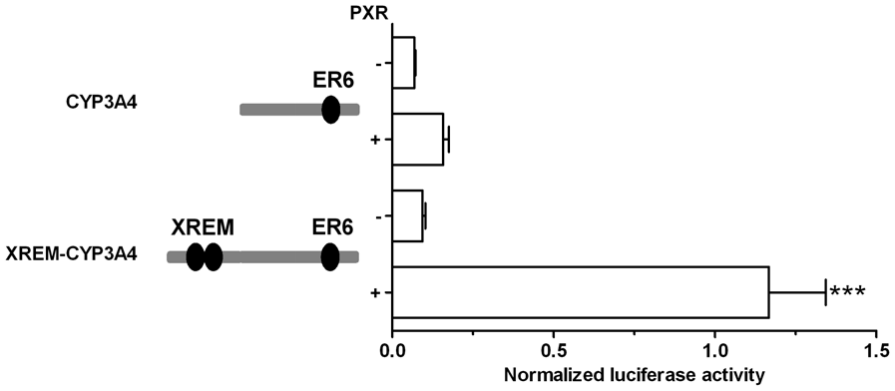
The effect of PXR overexpression on the *XREM-CYP3A4*-driven luciferase activity in MDCK.2 cells. The wild-type 374 bp *CYP3A4* and the chimeric *XREM-CYP3A4* constructs were transiently transfected in MDCK.2 cells. (+) and (−) indicate transfection with a PXR-expressing plasmid and with the same empty plasmid, respectively. Data are expressed as mean values (± SEM) of three to five independent experiments conducted as triplicates. Promoter-driven firefly luciferase activities in the individual wells were normalized using activities of the co-transfected renilla luciferase driven by a constitutive promoter. Statistically significant differences are indicated by asterisks (*** *p*<0.001).

We then addressed the importance of the transcriptional *CYP3A* regulator PXR, which is expressed in the small intestine, but not in the kidney [12], [28], [29], [30], [31], [32], [33]. We hypothesized that PXR may offset the inhibitory effect of YY1 on the CYP3A4 expression in the small intestine. In this case, a similar effect could reasonably be expected from renal cells transfected with PXR. However, the co-transfection of a PXR-expressing construct had only a weak (two-fold increase) and statistically not significant effect on the activity of the proximal *CYP3A4* promoter (Figure 8). We then co-transfected into these cells PXR together with the proximal *CYP3A4* promoter extended by the PXR-responsive enhancer XREM present in the *CYP3A4*, but not in the *CYP3A5* distal promoter [34]. In this case, PXR resulted in a 13-fold increase in the luciferase activity (Figure 8). Notably, the XREM inclusion had no effect on the luciferase activity in the absence of PXR co-transfection.

### Differential induction of CYP3A5 in mouse tissues

The above observations were consistent with a PXR-regulated expression of *CYP3A5* (as well as of CYP3A4) in the small intestine, and with a PXR-independent *CYP3A5* expression in the kidney. We hypothesized that these relationships would result in a differential response of *CYP3A5* in these organs to typical PXR agonists *in vivo*. This was investigated in mice transgenic for firefly luciferase driven by a 6.2 kb fragment of the human *CYP3A5* proximal promoter. A detailed analysis of the strains generated by two independent transgenic founders will be presented elsewhere. The luciferase activities were similar in both strains and sexes and the tissue distribution largely reflected that of *CYP3A5* transcripts in humans. The highest luciferase activity was detected in the small intestine, followed by organs without PXR expression [12], [28], [29], [30], [31], [32], [33] such as lung, adrenal gland, ovary, testis, prostate, and kidney (Figure S3). In addition, luciferase was detected in the forestomach, a structure absent in humans, and in the adjacent oesophagus.

**Figure 9:**
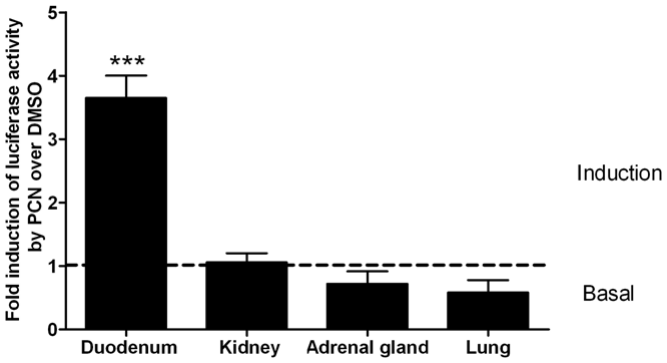
The effect of PCN on the expression of firefly luciferase driven by 6.2 kb of a human *CYP3A5* promoter in the duodenum, kidney, adrenal gland, and lung of transgenic mice. Mice (3 males and 3 females per treatment group) were injected i.p. with PCN (50 mg/kg) or the DMSO solvent. Organ homogenates were assayed with luciferase reporter gene assay (Promega) using a luminometer. Data are represented as ratio of RLU per µg protein of PCN over DMSO, shown as mean values (± SEM). Statistically significant differences are indicated by asterisks (*** p<0.001).

Transgenic mice of either sex were injected i.p. with 50 mg/kg of the agonist of the murine PXR pregnenolone-16α-carbonitrile (PCN) or with the dimethylsulfoxid (DMSO) solvent. Mice were sacrificed by cervical dislocation 24 hours after treatment and luciferase activities were determined in the homogenates of the kidney, lung, adrenal gland, and of the duodenal part of the small intestine. The *CYP3A5* transgene was 3.7-fold induced by PCN in the duodenum, whereas no induction was observed in the three organs lacking PXR (Figure 9). In contrast to human kidneys [12], [28], [29], [30], [31], [32], [33], mouse kidneys may express low levels of *PXR* transcripts [32], [44], although there are reports to the contrary [45], [46]. To minimize the risk of overlooking the *CYP3A5* induction in this organ, we exposed our transgenic mice to a still higher PCN dose of 100 mg/kg. The induction in the duodenum increased to 7-fold, but it was still absent from the kidney. Similarly to the 50 mg/kg dose, we observed no sex-dependent differences in the PCN response (data not shown).

## Discussion

Considering the expression of CYP3A5 in several steroidogenic organs, reports of its induction by PXR seemed paradoxical, as enhanced CYP3A5 activity could affect the steroid homeostasis. Admittedly, it has been noticed that aside from liver and small intestine, CYP3A5 is expressed exclusively in organs devoid of PXR expression [12], so that induction could be restricted to the former two organs. However, this in turn raises questions about the mechanism of CYP3A5 expression outside liver and small intestine, as the importance of PXR in CYP3A regulation is paramount. In the present work we illuminate these issues by demonstrating that the expression of CYP3A5 in most organs expressing this enzyme is indeed independent from PXR and in consequence irresponsive to the latter one's ligands, at least in transgenic mice. This constitutes a first description of uncoupling induction from constitutive expression for a major detoxifying enzyme, and of the underlying mechanism.

The expression of CYP3A5 in organs lacking PXR appears to be enabled by the loss of a suppressive YY1-binding element from the *CYP3A5* promoter during primate evolution. To arrive at this conclusion, we applied a combination of in vitro, in vivo, and transgenic techniques. We first established a two-cell line model of the small intestine and kidney, two organs expressing CYP3A5. The LS174T cells have been repeatedly validated as a faithful model of the basal and drug-induced CYP3A expression in the small intestine [34], [47], including the induction of CYP3A5 [2]. The MDCK.2 cells exhibit many characteristics of the collecting duct cells, a principal site of CYP3A5 expression in the kidney [16], [17], [18]. Transfected with *CYP3A4* and *CYP3A5* promoter constructs, these cell lines fully reflected the expression relationships between these genes in the kidney and small intestine [12], with both genes expressed at similar levels in small intestinal, but only CYP3A5 in renal cells.

The importance of YY1 in the expression of CYP3A5 in renal cells is supported by several lines of complementary evidence obtained from mutated *CYP3A5* promoter constructs, from comparisons to the *CYP3A4* promoter, and from chimeras comprising elements of both promoters. Thus, the *CYP3A4*-derived 57 bp fragment comprising a consensus YY1-binding site inserted into the *CYP3A5* promoter inhibited its transcriptional activity in renal cells. Conversely, its deletion from the *CYP3A4* promoter derepressed the luciferase activity in the same cell line. The specific involvement of YY1 in these effects was demonstrated by the effects of its mutagenesis, which mimicked the transcriptional effects of the entire 57 bp fragment. Thus, mutations designed to disrupt the YY1-binding site increased the activities of the *CYP3A4* and *CYP3A5* promoters, whereas optimizing the core sequence of the YY1 site had an opposite effect. Lastly, this sequence bound YY1 [39], as confirmed in our study.

YY1 is a ubiquitously expressed and evolutionary conserved member of the GLI-krüppel family of zinc finger transcription factors [43], which have been implicated in the transcriptional regulation of numerous genes important for cell proliferation, differentiation, and metabolism [48]. Depending upon the promoter context, YY1 can function either as a transcriptional activator or repressor [43], with the last-mentioned function apparently applying to *CYP3A*. YY1 may repress transcription directly, indirectly via cofactor recruitment or displacement, or via conformational DNA changes [49] and the elucidation of the exact mechanism applying to *CYP3A* requires further detailed studies.

The *CYP3A* YY1 binding site predates primate origin and its suppressing function seems to be conserved across primates, as demonstrated by a comparison of the ortholog elements from human and galago. We speculate that this regulatory element originally may have helped to restrict the tissue spectrum of CYP3A expression. This may have been important for the homeostasis of endobiotics such as steroid hormones, some of which (testosterone, corticosterone, progesterone and androstenedione) are proven CYP3A substrates [1], [21], [50], [51]. The YY1 binding site was deleted from the *CYP3A5* gene lineage together with additional sequence altogether comprizing 57 bp of the promoter sequence. This deletion occurred early in *Haplorrhini* following the separation from *Strepsirrhini* via one of two alternative two-step scenarios (Figure 4A). In one scenario, the first step comprised the more distal 25 bp and occurred in the common ancestor of *Tarsiiformes* and *Simiiformes* (i.e. some 57 million years ago), as indicated by a 25 bp deletion found in one of the two tarsier genes. Following the separation of *Tarsiiformes* and *Simiiformes*, the more proximal part was subsequently lost in a common ancestor of the latter primate infraorder. This occurred not later than 40 million years ago, since the 57 bp deletion is detected in both parvorders of *Simiiformes*, i.e. in Old World monkeys (human, chimpanzee, rhesus), and in New World monkeys represented by the marmoset. The second scenario comprises two independent deletions of different lengths, but of the same distal boundary occurring in *Tarsiiformes* and *Simiiformes* following their separation (Figure 4A). In either case, the 57 bp fragment was lost from the entire *CYP3A5* gene repertoire and not inserted into the human *CYP3A4* promoter, as suggested previously by a comparison of exclusively human *CYP3A4* and *CYP3A5* promoter sequences [27], [52]. The 10 bp deletion partly overlapping with the 57 bp deletion found in the chimpanzee *CYP3A67* was apparently an unrelated event, as judged from the intact sequence in the corresponding region in its closest paralog genes, i.e. *CYP3A7*.

Based on the cell line data we predicted a differential response of *CYP3A5* in the kidney and small-intestine to PXR-driven induction. We reasoned that since in the mouse *PXR* is strongly expressed in the small intestine but at best weakly in the kidney [32], [44], the *CYP3A5* promoter activity would be enhanced by PXR agonists in the former, but unaffected in the latter organ. This prediction was verified and confirmed in mice expressing firefly luciferase under the control of a *CYP3A5* promoter fragment. For mouse transgenesis we used a larger (6.2 kb) CYP3A5 promoter fragment to maximize the chances to recapitulate the CYP3A5 tissue expression in humans. Indeed, while our cell line data suggest that the loss of YY1-mediated repression was necessary for CYP3A5 expression in organs lacking PXR such as the kidney, this loss could not be the only determinant of the CYP3A5 organ expression, as this expression is ubiquitous neither in humans nor in our transgenic mice. While the identification of other determinants of the CYP3A5 tissue expression spectrum will require further studies, most of them are bound to be contained within the 6.2 kb *CYP3A5* promoter fragment. This is indicated by the striking similarity between the tissue distribution of the luciferase in our transgenic mice and the CYP3A5 expression in humans. The only major difference is the absence of luciferase expression in the liver, which suggests the existence of a liver-specific enhancer outside the promoter fragment used for transgenesis. There is increasing evidence that gene clusters are co-regulated [53] and it is tempting to speculate that the liver expression of CYP3A5 may require an enhancer shared with the other *CYP3A* genes, which form a cluster on chromosome 7.

The differential changes in luciferase activity in the kidney and small intestine in response to the mouse PXR agonist PCN is in agreement with the observations by Cheng and Klaassen, who detected an intestinal, but not renal, induction of the mouse gene *Cyp3a11* in response to the same compound [44]. Since the *PXR* expression in human kidneys is either non-detectable or at least much lower than in mouse kidneys, we infer that *CYP3A5* in human kidneys is similarly irresponsive to PXR activators. This is consistent with the failure of the agonist of the human PXR rifampicin to affect the renal activity of the PXR target P-glycoprotein in human subjects [54]. In turn, the small-intestinal induction of *CYP3A5* in our transgenic mice in response to PCN is in agreement with the upregulation of this gene in small intestines of humans treated with the agonist of the human PXR rifampicin [2].

Besides the kidney, *CYP3A5* induction was also absent from the adrenal gland and lung, i.e. tissues, which in humans and mice exhibit none or at best a very low level of *PXR* [12], [28], [29], [30], [31], [32], [33], [45]. This suggests that the *CYP3A5* expression in human organs unrelated to xenobiotic response (i.e. other than small intestine and liver) may be generally irresponsive to PXR-mediated induction, as already demonstrated for the kidney [54]. Furthermore, we speculate that the loss of the YY1-mediated transcriptional repression may have enabled the constitutive CYP3A5 expression in all organs expressing this enzyme aside from liver and small intestine. This speculation is strongly supported by the findings by Biggs et al. [27], which provided one of the starting points and many experimental ideas for our investigation. These workers demonstrated a derepression of a CYP3A5 promoter activity in a lung-derived cell line upon deletion of the same 57 bp fragment as in our study. The loss of the YY1-mediated transcriptional repression may have thus allowed for the widening of the CYP3A5 tissue expression in the absence of induction. This has allowed on the one hand, for avoiding the deleterious effects of *CYP3A5* induction on the homeostasis of any endogenous substrates of the CYP3A5 protein, such as steroids. On the other hand, the CYP3A5 expression outside the liver and small intestine must have conferred fitness advantages, which remain to be identified. Renal *CYP3A5* expression may have enhanced salt and water retention mediated by CYP3A5-catalyzed 6β-hydroxycortisol, which may have been advantageous in a hot climate. This mechanism has been suggested to be responsible for the high prevalence of the gene polymorphism-driven CYP3A5 expression in Africans, most of which express CYP3A5 in the kidney, perhaps at the expense of an increased risk of salt-dependent hypertension [55]. Taken together, the PXR-independent CYP3A5 expression outside the liver and small intestine may have evolved in primates to employ this enzyme in endobiotic homeostasis protected against potentially deleterious effects of xenobiotic-driven induction. To our knowledge, this is a first evolutionary description of the mechanism uncoupling the inducible and constitutive expression in a major detoxifying enzyme. Similar mechanisms may have evolved for other detoxifying proteins, many of which metabolize endobiotics.

Although this work focuses on CYP3A5, some of our observations illuminate the regulation of CYP3A4, which is expressed concomitantly with CYP3A5 in the liver and small intestine. Considering the ubiquitous expression of YY1, the presence of a transcriptionally repressive YY1 element in the CYP3A4 promoter seemed to be at odds with the expression of *CYP3A4* in these organs. Subsequent experiments designed to resolve this contradiction suggest that the inhibitory effect of YY1 on *CYP3A4* promoter activity is overridden, at least in small-intestinal cells, by the concerted action of one *trans*- and one *cis*-acting factor. We have identified these factors using MDCK.2 cells, which normally do not support *CYP3A4* expression, due to the inhibitory effect of the YY1 on its promoter. Through co-transfection of the transcriptional CYP3A regulator and xenobiotic sensor PXR, we conferred onto these cells a capability to express *CYP3A4*. PXR is normally expressed in the small intestine, but not in the kidney [12], [28], [29], [30], [31], [32], [33]. This suggests that the expression of PXR, acting in *trans*, is an indispensable determinant of the CYP3A4 expression in organs such as small intestine.

Besides PXR, the expression of *CYP3A4* in MDCK.2 cells required the presence of the PXR-responsive, *cis*-acting element XREM, located in the distal part of the *CYP3A4* promoter. Together with the proximal ER6 (Figure 2) and the far-distal constitutive liver enhancer module (CLEM), XREM represents the original scheme of CYP3A regulation by nuclear receptors such as PXR in placental mammals [34]. The need to offset the inhibitory effect of YY1 may have been the force driving both the conservation of XREM and the origin of novel PXR-responsive elements outside XREM recently described in the *CYP3A4* gene lineage [34]. Conversely, the loss of XREM from the *CYP3A5* gene lineage [34] is consistent with the reduced pressure to maintain XREM, conferred by the loss of the transcriptionally repressive YY1 binding site. In support of this interpretation, the losses of the YY1 binding element (Figure 4A) and of XREM [34] from the CYP3A5 gene lineage occurred simultaneously in evolutionary terms, since they are restricted to *Haplorrhini*.

The XREM-mediated, *CYP3A4* expression-promoting effect of PXR may have been additionally facilitated by the apparent attenuation of the YY1 inhibitory effect. This attenuation is conferred by the mutation of the YY1 consensus site core sequence CAT>CAC, which is present in all *Haplorrhini CYP3A* genes containing this element, except the pseudogene *CYP3A43*. The importance of this mutation was suggested by the diminished score values and confirmed by mutagenesis. The results of this latter experiment suggest that the sequence change in the YY1 core sequence may contribute to the high expression level of CYP3A4 in humans. This mutation may contribute to the differential expression of CYP3A4 and CYP3A5 in the small intestine and kidney in humans, acting in concert with the loss of the YY1 binding element from the *CYP3A5* promoter together with the differential organ expression of PXR and the higher accumulation of ancestral PXR response elements in *CYP3A4*.

We are aware of several shortcomings of our investigations. For example, transient transfections may not adequately recapitulate gene regulation in a natural chromatin context. On the other hand, both our cell line-derived data as well as those by Biggs and colleagues [27] are fully consistent with the *CYP3A5* organ expression and with the response to PXR activators in transgenic mice and in selected human organs such as small intestine, liver [2], and kidney [54]. Likewise, our results from transgenic mice do not formally prove the role of YY1 in the differential expression of CYP3A4 and CYP3A5 in human organs. They were conducted primarily to test the prediction of the differential organ induction of CYP3A5. However, this role is strongly suggested by the accumulating data on the effects of the YY1 site on promoter activity in cell lines derived from three relevant human organs (lung [27], small intestine, and kidney). Taken together, YY1 formally affects the activity of CYP3A promoters analyzed in cell lines. However, its effects are fully consistent with the available information on the differential organ expression and induction of CYP3A5 and CYP3A4 *in vivo*.

## Materials and Methods

### Chemicals

PCN and DMSO were obtained from Sigma (St. Louis, MO). D-Luciferin was purchased from BD Gentest (Woburn; MA). All other chemicals used in this study are commercially available molecular biology grade.

### Cell Culture, Transient Transfection and Luciferase Reporter Gene Assay

Human colon carcinoma-derived LS174T cells [56] and the canine kidney-derived MDCK.2 cells [57], were obtained from American Type Culture Collection. Both cell lines were maintained as described for LS174T cells [58], except that the MDCK.2 cell culture medium lacked the 1% essential amino acid supplement. LS174T and MDCK.2 cells were transfected using the Gene Juice transfection reagent (Novagen) and luciferase activities measured as described [59]. In PXR transactivation experiments 10 ng of the plasmid pcDhuPXR [60] were co-transfected.

### CYP3A Promoter Sequence Analysis

Approximately 1 kb of CYP3A sequence upstream from exon 1 from human (Homo sapiens), rhesus (Macaca mulatta), and chimpanzee (Pan troglodytes) was downloaded via Ensemble genome browser or NCBI Genbank. The corresponding sequences from marmoset (*Callithrix jacchus*) CYP3A5, CYP3A21 and CYP3A90, and the galago (Otolemur garnetti) CYP3A91 and CYP3A92 genes were obtained from bacterial artificial chromosome (BAC) sequences (CH259-48H24 and CH256-241K21), respectively [61]. Tarsier (Tarsius syrichta) sequences were identified from the whole-genome shotgun sequence database in NCBI using BLASTn [62]. Sequence alignment was performed using Multi-LAGAN [63] and visualized in BIOEDIT [64]. P-Match [65] was used for identification and scoring of YY1 DNA response elements. Matching was performed to predefined vertebrate matrices in a liver specific profile.

### Construction of Reporter Gene Constructs

The proximal 370 bp of the CYP3A5 promoter were amplified from the BAC clone 22300 [66] with *Nco*I- and *Nar*I-extended primers (Table S1). The digested and gel-extracted (Gene Jet gel extraction kit, Fermentas) PCR product was ligated to the analogously digested pGL3-Basic vector (Promega). CYP3A5-luc transgenic mice were generated via pronuclear injection of a plasmid expressing firefly luciferase under the control of the proximal 6.2 kb of the human CYP3A5 promoter. To this end, a 5.4 kb and a 555 bp fragment of the human CYP3A5 promoter were amplified from the BAC clone 22300 with *Mlu*I/*Nco*I and *Kpn*I/*Avr*II extended primers (Table S1), sequentially sub-cloned into the CYP3A5-370 construct and confirmed by sequencing. The 374 bp *CYP3A4* promoter construct and the chimerical XREM-CYP3A4 construct were described previously [58], [67].

### Inverted PCR-based Mutagenesis

The insertions and deletions into the wild-type *CYP3A5* and *CYP3A4* promoter constructs were generated using an inverted PCR-based method as described [27], using primers listed in Table S1. Insertion primers were 3′-complementary to the template plasmid and 5′-extended by the sequence to be inserted. PCR products amplified with the High Fidelity Taq polymerase (Bioline) were subjected to *Dpn*I digestion (Fermentas) to remove the dam-methylated parental templates. After purification on a column (Fermentas), PCR products were further digested with Mung Bean exo-nuclease (New England Biolabs) for 90 minutes to obtain blunt ends for the subsequent ligation using a T4 ligase (New England Biolabs).

### Site-directed Mutagenesis of the YY1 Binding Site

All mutations were introduced into the CYP3A5-57ins and CYP3A4-374 constructs using the QuikChange Site-Directed Mutagenesis Kit (Stratagene), according to manufacturer's instructions, and primers listed in Table S2. All clones used in transfection experiments were confirmed by sequencing.

### Nuclear Extract Preparation

Confluent MDCK.2 cells were washed twice with ice-cold PBS, and detached using a rubber policeman in 1 ml of the hypotonic buffer A, consisting of 10 mM HEPES (pH 7.9), 10 mM KCl, 0.1 mM EGTA, 0.1 mM EDTA, 0.1% benzoase (Novagen) and 2% EDTA-free protease inhibitor cocktail (Calbiochem). Cells were pelleted at 750 g for 5 min, resuspended in 1 ml of buffer A with 0.4% IGEPAL (Sigma) and kept on ice for 15 min for cell swelling and membrane lysis. After gentle centrifugation, the nuclear pellet was resuspended in 100 µl of an ice-cold hypertonic buffer (Buffer B: 20 mM HEPES (pH 7.9), 0.4 M NaCl, 1 mM EDTA, 1 mM EGTA, 1 mM dithiothreitol, 0.1% benzoase, 2% EDTA-free protease inhibitor cocktail) and vigorously mixed for 60 minutes at 4°C to disrupt the nuclear membrane. This was followed by centrifugation at 7,500 g for 15 minutes at 4°C to remove nuclear debris. The supernatant (nuclear extract) was collected and stored in aliquots at −80°C. Protein concentration was determined by the Bradford method. The enrichment of nuclear proteins was confirmed by Western blot using antibodies against a nucleus-specific (lamin B) and a cytosol-specific (GAPDH) protein (data not shown).

### Electrophoretic Mobility Gel Shift Assay

A nucleotide containing the (underlined) YY1 consensus binding site from the CYP3A4 promoter (ttggaagaggcttctccaccttggaagttggca), a positive YY1 control (cgctccgcggccatcttggcggctggt), and the respective complement oligonucleotides were 5′-labelled with IRDye 800 (Metabion). The positive control contains a previously reported YY1-binding site [41]. Equimolar amounts of complementary oligonucleotides were annealed by boiling for 5 minutes at 100°C followed by slow cooling to room temperature. Thus obtained double-stranded labeled probes were diluted with double-desalted water and stored in aliquots at −20°C in light-protected tubes until use. EMSA reactions contained 10 mM HEPES (pH 7.9), 60 mM KCl, 0.2% IGEPAL (Sigma), 6% Glycerol, 2 mM dithiothreitol, 1 µg poly d(I-C) (Sigma), 20 µg of nuclear extract, 50 fmol of a ID800-labeled probe in a total volume of 10 µl. Supershift reactions additionally included 1 µg of the anti-YY1 (sc-7341×) or 300 ng of the anti-PXR (sc-7737) antibodies (Santa Cruz Biotechnology). Reactions were pre-incubated 15 minutes or, for supershift, 30 minutes at room temperature and incubated for further 20 minutes after the addition of the labeled probe in a volume of 2.5 µl. Samples were subsequently resolved by native PAGE in a pre-run 4% minigel in 0.5× TBE at 100 Volt for 60 minutes at 4°C and visualised with an Odyssey infrared imager (LI-COR Biosciences) with focus offset at 0.375 mm.

### Generation of CYP3A5-luciferase Transgenic Mice

Two transgenic lines were established by pronuclear injection of a plasmid expressing firefly luciferase under the control of the proximal 6.2 kb of the human *CYP3A5* promoter (see above). Two founders were identified by Southern blotting (data not shown). The transgene was kept on the original genetic background C57BL/6J by breeding heterozygous carriers with wild-type C57BL/6J mice. Transgenic mice were identified by PCR of genomic DNA isolated from mice tail tips, using primers CYP3A5-Fw and CYP3A5-Rv primers listed in Table S1, which generate a 500 bp PCR amplicon. Transgenic and wild-type mice were housed in our animal facility and maintained under controlled environmental conditions with a 12 h/12 h light/dark cycle. Food and water were available *ad libitum*.

### Transgenic Mice Treatment

All animals experiment described in this study were approved by the responsible animal ethics committee. Male and female 6 to 8 weeks old transgenic mice weighing 20 to 29 g were used to determine the effect of PCN on the CYP3A5-luc transgene activity in the duodenum and kidney in vivo. Mice (n = 3 per group and sex) were injected i.p. with 50 or 100 mg/kg of the murine PXR agonist PCN dissolved in DMSO. Control mice were injected with DMSO only. Mice were sacrificed by cervical dislocation 24 hours after the treatment. Tissue samples were rapidly removed, washed in ice-cool 1× PBS, snap-frozen in liquid nitrogen, and stored at −80°C until used. Tissues were homogenized by at least ten strokes of a tissue disrupter (Ultraturrax) in 200–500 µl of a cell lysis buffer (Promega). Homogenates were shock-frozen in liquid nitrogen, thawed and subsequently centrifuged at 5000 g for 5 minutes at 4°C. The supernatant was collected for measurement of luciferase activity. Protein concentration was determined by the Bradford method. Luciferase activity was determined as described above and expressed as relative light units (RLU) per µg of total protein.

### Statistical Analyses

Statistically significant differences were calculated with Mann-Whitney U test or one-way ANOVA with a Dunnett's post-test if applicable. All tests were performed using GraphPad Prism version 5.0 (GraphPad Software, San Diego, CA). Results were considered as statistically significant at P values<0.05.

## Supporting Information

Figure S1 **Mutational analysis of the NF1 and the E-box in** ***CYP3A4*** **and** ***CYP3A5-57ins*** **constructs in MDCK.2 cells.** The mutations either restore the NF1 consensus core motif (*CYP3A5-ins57NF1/SP*) or disrupt the NF1 (*CYP3A4-NF1M*) or the E-box site (*CYP3A4-EboxM* and *CYP3A5-57insEboxM*). Mutants, wild-type CYP3A4 (A), and CYP3A5 (B) promoter constructs were transiently transfected into MDCK.2 cells. Promoter-driven firefly luciferase activities were normalized using activities of the co-transfected renilla luciferase driven by a constitutive promoter and compared to that of the wild type construct. Data are expressed as mean values (±SEM) of four independent experiments conducted as triplicates. Statistically significant differences are indicated by asterisks (***p<0.001).
(TIF)

Figure S2 **The effect of YY1 overexpression on the** ***CYP3A4*** **-driven luciferase activity in LS174T cells.** The wild-type 374 bp *CYP3A4* construct was transiently transfected in LS174T cells. (+) and (**−**) indicate transfection with an YY1-expressing plasmid and with the same empty plasmid, respectively. Data are expressed as mean values (± SEM) of eight independent experiments conducted as triplicates. Promoter-driven firefly luciferase activities in the individual wells were normalized using activities of the co-transfected renilla luciferase driven by a constitutive promoter. The statistically significant difference is indicated by asterisks (*** *p*<0.001).
(TIF)

Figure S3 Tissue distribution of the luciferase activity of the *CYP3A5-luc* transgene**.** Organs were isolated from transgenic mice (n = 4 per group) from line A (TGA) and line C (TGC). Organ homogenates were assayed with a luciferase reporter gene assay (Promega) using a luminometer. Data from female (A) and male (B) are relative light units (RLU)/µg protein, shown as mean values ±SEM.
(TIF)

Table S1 **Oligonucleotides used for cloning of the** ***CYP3A5*** **proximal promoter constructs (1 to 6), PCR genotyping of the transgenic mice (7 and 8), and for insertions and deletions (9 to 16).**
(PDF)

Table S2 **Oligonucleotides used for site-directed mutagenesis of the YY1 binding site.**
(PDF)
