# Supplementary figures and images for: Pregnane X Receptor and Yin Yang 1 Contribute to the Differential Tissue Expression and Induction of CYP3A5 and CYP3A4
Source: PLoS One. 2012 Jan 23;7(1):e30895. doi: 10.1371/journal.pone.0030895 (PMC3264657; doi:10.1371/journal.pone.0030895)

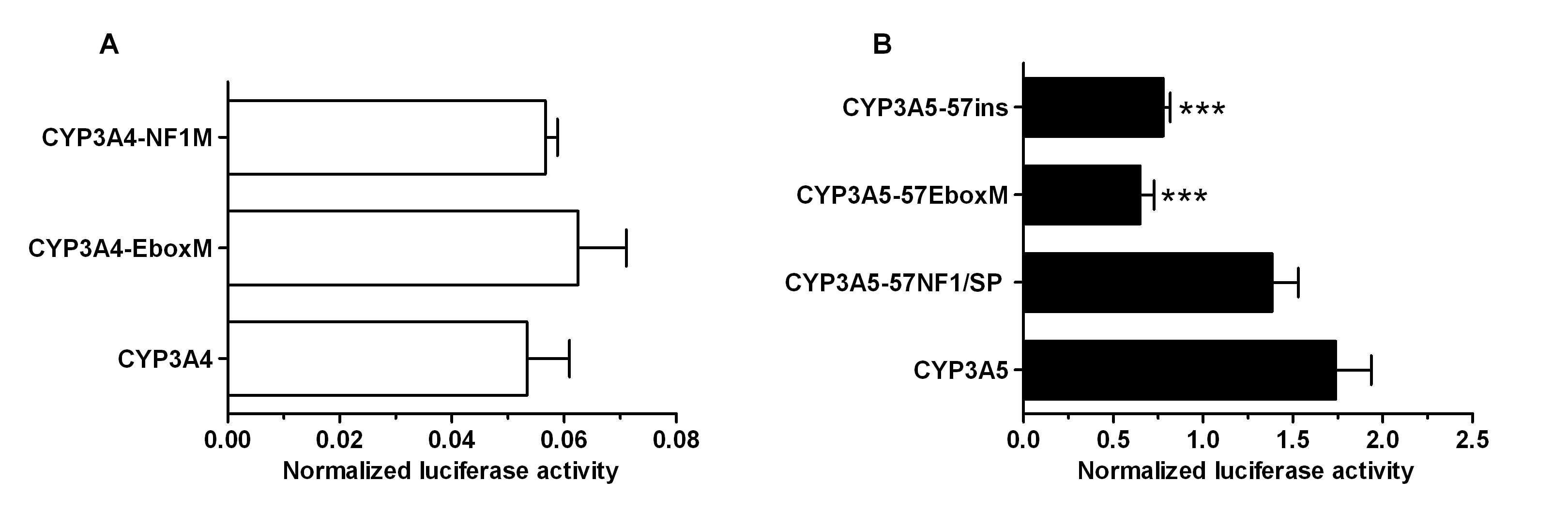

Supplement: Figure S1 — Mutational analysis of the NF1 and the E-box in CYP3A4 and CYP3A5-57ins constructs in MDCK.2 cells. The mutations either restore the NF1 consensus core motif (CYP3A5-ins57NF1/SP) or disrupt the NF1 (CYP3A4-NF1M) or the E-box site (CYP3A4-EboxM and CYP3A5-57insEboxM). Mutants, wild-type CYP3A4 (A), and CYP3A5 (B) promoter constructs were transiently transfected into MDCK.2 cells. Promoter-driven firefly luciferase activities were normalized using activities of the co-transfected renilla luciferase driven by a constitutive promoter and compared to that of the wild type construct. Data are expressed as mean values (±SEM) of four independent experiments conducted as triplicates. Statistically significant differences are indicated by asterisks (***p<0.001). (TIF) [file pone.0030895.s001.tif]

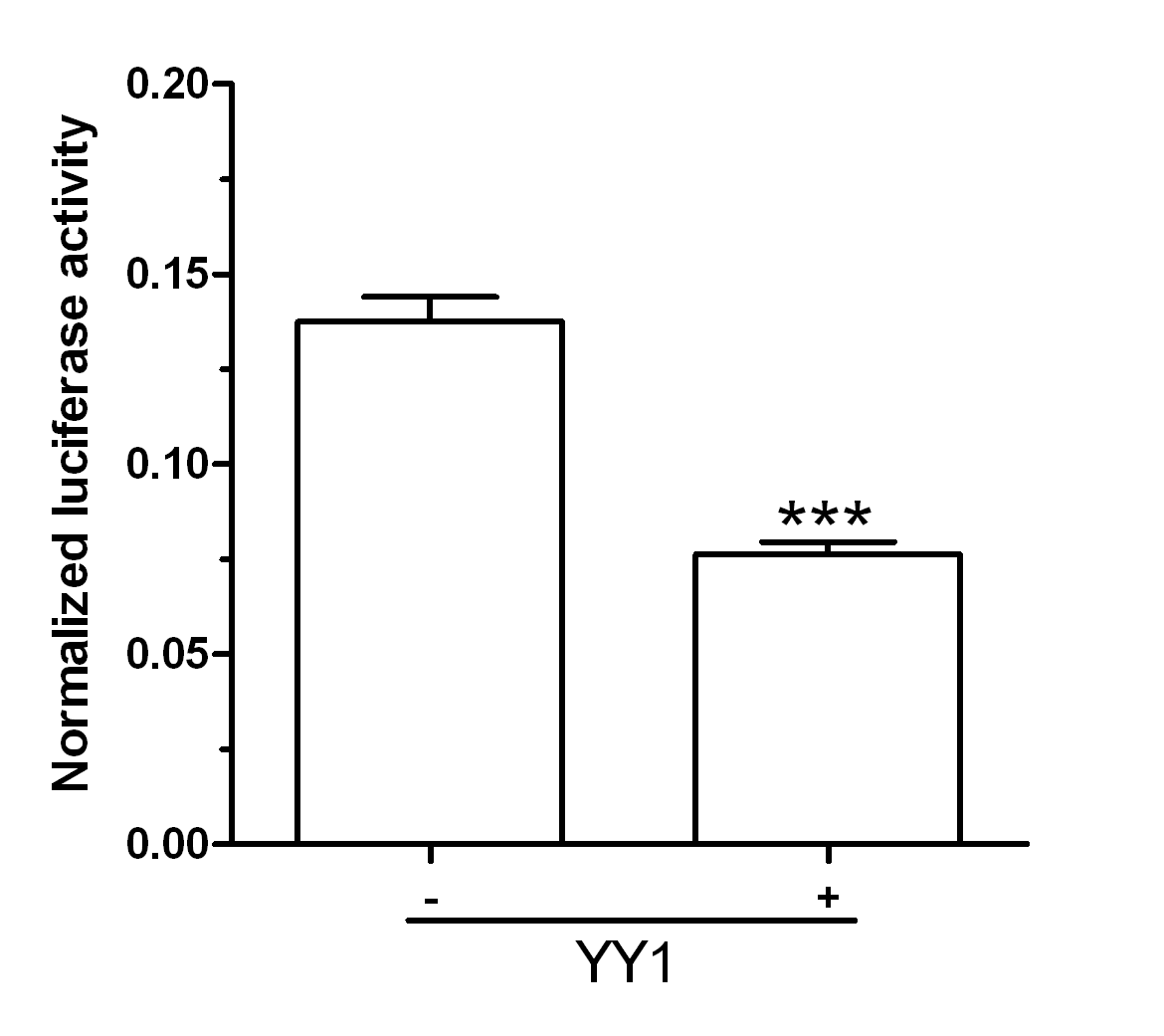

Supplement: Figure S2 — The effect of YY1 overexpression on the CYP3A4 -driven luciferase activity in LS174T cells. The wild-type 374 bp CYP3A4 construct was transiently transfected in LS174T cells. (+) and (−) indicate transfection with an YY1-expressing plasmid and with the same empty plasmid, respectively. Data are expressed as mean values (± SEM) of eight independent experiments conducted as triplicates. Promoter-driven firefly luciferase activities in the individual wells were normalized using activities of the co-transfected renilla luciferase driven by a constitutive promoter. The statistically significant difference is indicated by asterisks (*** p<0.001). (TIF) [file pone.0030895.s002.tif]

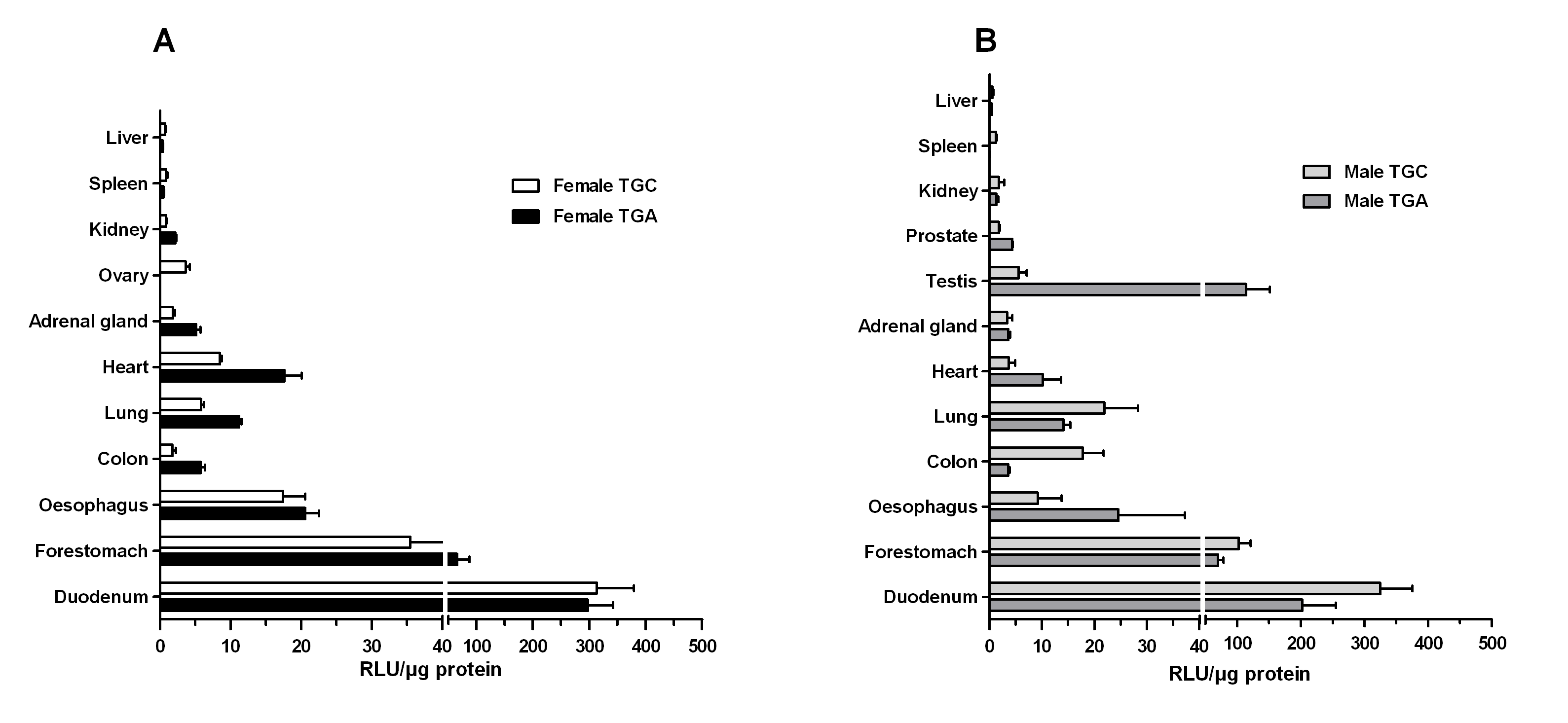

Supplement: Figure S3 — Tissue distribution of the luciferase activity of the CYP3A5-luc transgene. Organs were isolated from transgenic mice (n = 4 per group) from line A (TGA) and line C (TGC). Organ homogenates were assayed with a luciferase reporter gene assay (Promega) using a luminometer. Data from female (A) and male (B) are relative light units (RLU)/µg protein, shown as mean values ±SEM. (TIF) [file pone.0030895.s003.tif]
