# Supplementary material for: Pregnane X Receptor and Yin Yang 1 Contribute to the Differential Tissue Expression and Induction of CYP3A5 and CYP3A4
Source: PLoS One. 2012 Jan 23;7(1):e30895. doi: 10.1371/journal.pone.0030895 (PMC3264657; doi:10.1371/journal.pone.0030895)
Supplement: Table S1 — Oligonucleotides used for cloning of the CYP3A5 proximal promoter constructs (1 to 6), PCR genotyping of the transgenic mice (7 and 8), and for insertions and deletions (9 to 16). (PDF) [file pone.0030895.s004.pdf]

Number	Primer name	Sequence (5’ to 3’)
**1**	CYP3A370-Fw	GGCAGCCATGGAGGGGCAGGTGAGAGG
**2**	CYP3A370-Rv	ATGGGCGCCGGGCCTTTCTTTATG
**3**	CYP3A5$_{5.4}$-Fw	CACAACTATCACAAACGCGTTGCGAAACC
**4**	CYP3A5$_{5.4}$-Rev	CCTCTCACCTGCCCCTCCATGGCTGCC
**5**	CYP3A5$_{6.2}$-Fw	GGCACAAAATGTATCCTAGGCTTATC
**6**	CYP3A5$_{6.2}$-Rev	GTGAGATGAACCCGGTACCTCAGATG
**7**	CYP3A5-Fw	GCCACCCCTAGTTAGCACC
**8**	cyp3a5-Rv	CTCGAACTCCTGACCTCAGG
**9**	CYP3A5-57ins-Fw	tgctacttccaactgcaggcagagcacaggtggcccTGCTATTGGCTGCAGCTATAGCCCTGCC
**10**	CYP3A5-57ins-Rv	aggcttctccaccttggaagttgGCAAAGAATCGCACACACCCCTTTGCTGACCTCTTTTGA
**11**	CYP3A5-57SP-Fw	cgaacgaacgaacgaacgaacgaacgaacgTGCTATTGGCTGCAGCTATAGCCCTGCC
**12**	*CYP3A5*-57SP-Rv	ttcgttcgttcgttcgttcgttcgttcGCAAAGAATCGCACACACCCCTTTGCTGACC
**13**	CYP3A4-57del/SPins-Fw	cgaacgaacgaacgaacgaacgaacgaacgTGCTACTGGCTGCAGCTCCAGCC
**14**	CYP3A4-57del/SPins-Rv	ttcgttcgttcgttcgttcgttcgttcAGTTGGCAAAGAATCACACACACACCACTC
**15**	*CYP3A4*-57del-Fw	TGCTACTGGCTGCAGCTCCAGCCCTGCCTCCTTCTCTAGC
**16**	*CYP3A4*-57del-Rv	GTTGGCAAAGAATCACACACACACCACTCACTGACCTCC

Nucleotides complementary to the respective promoter sequence are capitalized in each primer pair. Where appropriate, the circularization of the resulting PCR products by ligation was improved by 5’ phosphorylation of oligonucleotides.
