# Supplementary material for: Pregnane X Receptor and Yin Yang 1 Contribute to the Differential Tissue Expression and Induction of CYP3A5 and CYP3A4
Source: PLoS One. 2012 Jan 23;7(1):e30895. doi: 10.1371/journal.pone.0030895 (PMC3264657; doi:10.1371/journal.pone.0030895)
Supplement: Table S2 — Oligonucleotides used for site-directed mutagenesis of the YY1 binding site. (PDF) [file pone.0030895.s005.pdf]

**Primer name**	**Sequence (5’ to 3’)**
Canonical YY1 binding site	(C/g/a)(G/t)(C/t/a)**CAT**N(T/a)(T/g/c)
CYP3A5-57insM1/CYP3A4-M1	GTTGGAAGAGGCTTCTCCA**T**CTTGGAAGTTGGCAAAG
CYP3A5-57insM2	GTTGGAAGAGGCTTCTC**A**ACCTTGGAAGTTGGC
CYP3A5-57insM3	GGAAGAGGCTTCTCCACC**C**TGGAAGTTGG
CYP3A5-57insM4	GAGGCTTCTCCACC**GA**GGAAGTTGGCAAAG
CYP3A5-57insM5	GGAAGAGGCTTCTC**AG**CCTTGGAAGTTGGC
CYP3A5-57insM6	GGAAGAGGCTT**T**T**G**CACC**C**TGGAAGTTGG
CYP3A5-57insM7/CYP3A4-M7	GTTGGAAGAGGCTTCTC**AG**CC**GA**GGAAGTTGGCAAAG

Nucleotides corresponding to the canonical YY1-binding site are underlined. Mutated nucleotides are shown in bold type.
